# Luminescent peptide tagging enables efficient screening for CRISPR-mediated knock-in in human induced pluripotent stem cells

**DOI:** 10.12688/wellcomeopenres.15119.3

**Published:** 2019-07-11

**Authors:** Ralitsa R. Madsen, Robert K. Semple

**Affiliations:** 1 Centre for Cardiovascular Science, The Queen’s Medical Research Institute, University of Edinburgh, Edinburgh, EH16 4TJ, UK

**Keywords:** CRISPR, knock-in, human pluripotent stem cells, iPSCs, screening, HiBiT, tagging

## Abstract

Human pluripotent stem cells are increasingly used for CRISPR-mediated gene targeting in efforts to generate models of human diseases. This is a challenging task because of the high sensitivity of these cells to suboptimal conditions, including CRISPR-associated DNA damage and subsequent rounds of single-cell cloning. We sought to develop a sensitive method that enables rapid screening of CRISPR targeted cells, while preserving cell viability and eliminating the need for expensive sequencing of a large number of clones. A protocol was designed in which the luminescent peptide tag, HiBiT, is appended to the extracellular portion of an inert surface membrane protein (CD46), using synthetic CRISPR reagents and a widely distributed human induced pluripotent stem cell (iPSC) line. We find that this approach substantially reduces labour-intensive screening of CRISPR-targeted iPSCs and minimises the number of subcloning steps. Successfully edited iPSCs could be identified within a week of targeting, based only on extracellular luminescence detection in live cells. The total screening time in each round was less than 30 minutes and no sequencing was required. This method can be developed further to serve as a highly sensitive co-selection strategy in CRISPR knock-in experiments, particularly in the context of challenging cell lines.

## Introduction

The development of powerful gene editing technologies such as clustered regularly interspaced palindromic repeats (CRISPR)/Cas9, alongside continuously improving protocols for the derivation and maintenance of induced human induced pluripotent stem cells (iPSC), have made this cell type the model of choice in many settings, be it mechanistic disease studies or high-throughput screens aimed at identifying novel drug targets ^1^.

Gene editing in human iPSCs is particularly powerful when applied to generation of series of isogenic cell lines differing only with respect to a specific pathogenic mutation. The technology also enables tagging of endogenous proteins for studies that require differential cell labelling or the ability to pull down a target of interest. Nevertheless, such knock-in experiments remain challenging in human iPSCs, in part due to their sensitivity to CRISPR-induced DNA damage ^2^ and poor survival as single cells ^3^. Without *bona fide* selection, homology-directed repair (HDR) rates lower than 1 % are considered normal for human iPSCs and require picking of a relatively large number of individual colonies, or several rounds of sib-selection with subcloning ^4– 6^. This makes gene editing in human iPSCs costly because of expensive cell culture materials and the need for extensive sequencing of individual clones.

Faced with this challenge and the need to tag an extracellular surface protein for differential cell labelling, we sought to develop a method that allows for faster and cheaper screening of successfully targeted iPSCs, while minimising exposure to suboptimal culture conditions. Given the low HDR efficiency in human iPSCs, such a method would have to be highly sensitive in order to distinguish the presence of rare gene-edited cells in a mixed population consisting mainly of wild-type counterparts. Thus, we focused on adapting the HiBiT luminescence technology for efficient screening and co-selection of human iPSCs undergoing dual targeting. The HiBiT technology comprises a split version of the exceptionally bright NanoLuc luciferase. A small 11 amino acid peptide, HiBiT, can be fused to a protein of interest, and subsequent supplementation with the large subunit (LgBiT) results in strong binding to HiBiT, thereby reconstituting NanoLuc activity. CRISPR-mediated HiBiT tagging and luminescence detection were recently used to track HIF1 *α* dynamics in response to a range of stimuli, demonstrating the high sensitivity of this method in HEK293, HeLa and primary human umbilical vein endothelial cells ^7^. Importantly, the high sensitivity of the HiBiT system enables detection of very low amounts of target protein, down to femtomoles or lower ^7^.

A major advantage of this technology emerges when the tag is appended to the extracellular portion of a surface membrane protein because this allows luminescence detection of gene-edited cells without the need for cell lysis and DNA extraction for downstream PCR-based applications. Here, we demonstrate that tagging of the extracellular portion of the cell surface protein CD46 allows for efficient and cost-effective screening of CRISPR-targeted human iPSCs, reducing processing time to less than 30 minutes per round, eliminating the need for expensive genetic assays and minimising stress-inducing cell manipulations.

## Methods

### Routine cell culture

We used the male iPSC line WTC11 (Coriell # GM25256) due to its amenability to genome editing and known diploid karyotype. WTC11 whole-exome and wholegenome sequencing data are available via the Conklin lab website.

Prior to targeting, the cells were cultured in 6-well plates coated with hESC-qualified Geltrex (Thermo Fisher Scientific # A1413302) diluted 1:100 in DMEM/F12 (Sigma # D6421). Cells were maintained in Essential 8 Flex (E8/F) medium (Thermo Fisher Scientific # A2858501) and passaged every four days when 80-90 % confluent, with split ratios ranging from 1:10 to 1:15. ReLeSR (Stem Cell Technologies # 5872) was used to dissociate the cells nonenzymatically, and 1X RevitaCell (Thermo Fisher Scientific # A2644501) was included during the first 24 hours to promote survival.

### Targeting design

We chose the human protein CD46 (ENSEMBL gene id: ENSG00000117335) for tagging because it is mainly involved in autologous cell protection against the complement system as well as acting as a costimulatory factor for T-cells to promote CD4+ T cell differentiation (Uniprot P15529); it is thus unlikely that tagging of this protein’s extracellular portion will interfere with important biological functions of human iPSCs and most of their differentiated derivatives. We used our previously published RNAseq dataset to confirm that the gene is well-expressed in WTC11 ^6^.

CD46 is a single-pass type I membrane protein, with the N-terminus exposed on the extracellular side of the plasma membrane. The first 34 amino acids correspond to an endoplasmic reticulum (ER)-targeting signal sequence, which is cleaved from the mature protein. Consequently, the tag has to be knocked in after the signal peptide to avoid downstream removal. We used a published three-dimensional structure of CD46 ( PDB ID: 1CKL) to confirm that this region is not buried within the protein upon folding. According to our strategy, successful targeting would result in tagging of 15 of the 16 CD46 isoforms. A flexible triple Glycine (GGG) linker was inserted between the tag and the start of CD46’s extracellular portion. This linker cannot be cleaved by chymotrypsin, factor XA, thrombin or trypsin (checked against the SynLinker database).

For guide RNA design, the following 240 bp sequence was submitted to the CRISPRko module of the Broad Institute’s GPP web tool ^8^ (accessed 1 October 2018):

5’-TGCTATGAGCACTCAGGTAAAAGCATGGAACAGTCATTT AAAATCTTGCCAAGGGCCTTTCTGTTTTTTCTGTACTACCTGCTGCCAGACCACAGTCCATGGCTGATGAAAGTGATATCAGTACTTCATCTTCATGTTCCTATTCTCTTATCCCTAGATGCCTGTGAGGAGCCACCAACATTTGAAGCTATGGAGCTCATTGGTAAACCAAAACCCTACTATGAGATTGGTGAACGAGTA-3’

Two guide RNAs from the output list were selected based on their on- and off-target ranks as well as cut distance from the insertion site. One of these, annotated sgRNA_CD46_rank13 ( Table 1), was chosen for sub-sequent tagging experiments as it has previously been validated by IDT (Design ID: Hs.Cas9.CD46.1.AF).

**Table 1.** ALT-R XT CRISPR RNA (crRNA) and single-stranded oligo DNA (ssODN) sequences for CD46 targeting.

Description	Sequence
crRNA-CD46	5’-AAATGTTGGTGGCTCCTCAC-3’
ssODN- CD46-HiBiT	5’-GTACTACCTGCTGCCAGACC ACAGTCCATGGCTGATGAA AGTGATATCAGTACTTCATC TTCATGTTCCTATTCTCTTAT CCCTAGATGCCGTGAGCGGC TGGCGGCTGTTCAAGAAGAT TAGCggaggtggaTGTGAGGA GCCACCAACATTTGAAGCTA TGGAGCTCATTGGTAAACC AAAACCCTACTATGAGATTG-3’
ssODN- CD46-V5	5’-GTACTACCTGCTGCCAGACC ACAGTCCATGGCTGATGAA AGTGATATCAGTACTTCATC TTCATGTTCCTATTCTCTTAT CCCTAGATGCCGGTAAGCCTA TCCCTAACCCTCTCCTCGGTC TCGATTCTACGggaggtggaTG TGAGGAGCCACCAACATTTGAA GCTATGGAGCTCATTGGTAAAC CAAAACCCTACT-3’

Two 200 bp long HDR templates were designed manually, either including a sequence for HiBiT or a V5 tag ( Table 1), followed by the flexible GGG linker. All three templates were designed asymmetrically following established rules ^9^, using a shorter homology sequence to the PAM-distal region and complementarity to the nontargeted strand. Known WTC11-specific SNPs in the targeted CD46 region were taken into account following examination of the publicly available WTC11 whole-genome sequence. No silent mutations were introduced into the template because successful tagging destroys the PAM site, thus preventing repeated targeting. Using ExPASy’s PeptideCutter tool, we confirmed that the translated fusion products wouldn’t be targeted by intracellular proteases.

### Preparation of CRISPR/Cas9 targeting reagents

Single-stranded oligo DNA (ssODN) templates, ALT-R XT CRISPR RNA (crRNA), ALT-R trans-activating crRNA (tracrRNA) and ALT-R Cas9 Nuclease V3 were acquired from Integrated DNA Technologies (IDT).

Prior to use, the crRNA and the tracRNA were resuspended in pH 7.5 TE buffer (IDT # 11-01-02-02) to a final concentration of 100 *µ*M. To prepare crRNA:tracRNA duplexes at 50 *µ*M, equal volumes of each RNA were mixed and heated for 5 minutes at 95 °C, followed by controlled cool-off to 25 °C at ramp rate 0.1 °C/second. The formed duplexes were placed on ice until ready to use.

Ribonucleoproteins were prepared by mixing 5 *µ*l each of crRNA:tracRNA duplex (50 *µ*M) and recombinant Cas9 enzyme (61 *µ*M), followed by incubation at room temperature for 20 minutes. Next, 200 pmol of each HDR template was added to the RNPs prior to delivery into iPSCs.

### Nucleofection and luminescence-based screening of CRISPR-targeted iPSCs

Targeting was performed using healthy, subconfluent iPSCs (P51) pretreated with 1X RevitaCell (in E8/F) for 3 hours. The cells were dissociated with StemPro Accutase (Thermo Fisher Scientific # A110501) and 1e6 cells taken forward for nucleofection. Following low-speed centrifugation (100G, 3 minutes), the cell pellet was resuspended in 100 *µ*l nucleofection solution (P3 Nucleofection Kit, Lonza # V4XP-3024). Of this suspension, 85 *µ*l were transferred to the assembled RNPs, resulting in c. 850,000 cells in the final reaction. The nucleofection was carried out with an Amaxa 4D nucleofector, using programme CA137. Immediatley following nucleofection, 500 *µ*l E8/F with 1X RevitaCell were added to the cell suspension and transferred to a Falcon tube containing 4.5 ml E8/F with 1X RevitaCell. Of this suspension, 100 *µ*l were seeded into each well of a Geltrex-coated opaque-white Nunc 96-well plate (Thermo Fisher Scientific # 165306). The following day, the cells were replenished with 200 *µ*l fresh E8/F without RevitaCell and again on day 3 post-nucleofection.

Luminescence-based screening for successfully targeted cells was carried out 4 days post-nucleofection, using Promega’s Nano-Glo HiBiT Extracellular Detection System. Briefly, the iPSCs were washed once with 200 *µ*l Dulbecco’s PBS (DPBS, no calcium, no magnesium; Thermo Fisher Scientific # 14190144) per well, followed by addition of 50 *µ*l DPBS. Next, 50 *µ*l Nano-Glo HiBiT and LgBiT mixture were added to each well, followed by mixing and detection of the luminescent signal within 10 minutes, during which time the plate was kept away from light. Luminescence was detected on a BMG Clariostar, using top reading with lid to preserve sterility. The focal height was set to 11, and integration time was 1 second. Immediately following detection, the cells were transferred back to the biosafety cabinet and the solution removed from each well. Following a single wash in 200 *µ*l DPBS, each well was replenished with 200 *µ*l fresh E8/F. Although cell survival was not compromised with this procedure, we recommend the use of PBS with Ca ^2+^/Mg ^2+^ in future repeats as we noted that prolonged exposure to DPBS caused transient dissolution of cell-cell contacts within individual colonies.

Following recovery for 1-2 days, cells with a positive luminescence signal were expanded non-enzymatically (using ReLeSR) into a Geltrex-coated 24-well plate, prior to a round of subcloning into Geltrex-coated 96-well plates, seeding 12.5 cells/well in E8/F supplemented with 1X RevitaCell. From previous work, we know that this seeding density typically results in survival of 2-3 cells in each well and allows for efficient enrichment while minimising cell loss ^6^. RevitaCell was removed 6 days post-subcloning and the cells processed for a second round of luminescence-based screening once most wells in a plate contained at least one colony with diameter *>* 1000 *µ*m.

### PCR-based detection of CRISPR-mediated CD46 tagging

Genomic DNA (gDNA) was extracted from luminescence-positive cells before subcloning and was used for PCR amplification of the CRISPR-targeted CD46 locus (F primer: 5’-AAGTCCCATTTCCTCCACTAC-3’; R primer: 5’-ACAAGAAGAAAATCATCATCACCG-3’). The PCR was carried out using 50-100 ng gDNA and GoTAQ G2 Colourless Master Mix (Promega) with the following thermocycling conditions (Techne Prime Thermal Cycler): 95 °C for 1 minute, 30 cycles of 95 °C for 30 seconds - 60 °C for 20 seconds - 72 °C for 45 seconds, 72 °C for 45 seconds, 4 °C until collection. A FAM-labelled forward primer was used to enable detection of the PCR products (diluted 1:20) by capillary electrophoresis. This was carried out on a LabChip GX24 Nucleic Acid Analyser (Perkin Elmer) using the DNA 1K Reagent Kit (Perkin Elmer #CLS760673) according to the manufacturer’s instructions. Of note, prior to analysis, the PCR products were diluted 1:20 in nuclease-free water; this causes slower migration of the fragments due to low ionic strength. The correct size of the fragments was confirmed by conventional agarose gel electrophoresis (raw gel image included via OSF ^10^). We suggest dilution in TE buffer in future repeats.

## Results

**Figure 1.**
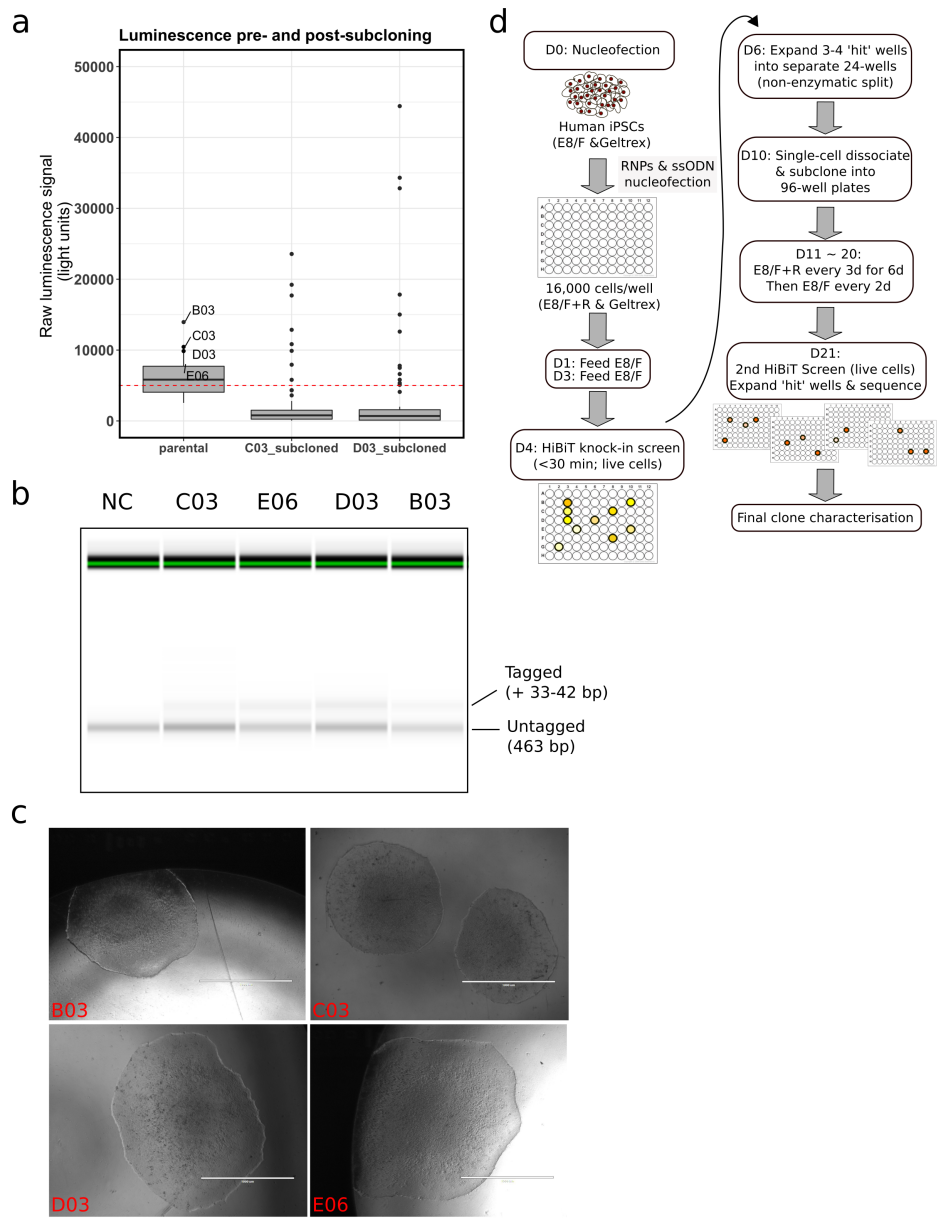
HiBiT-tagging of the membrane surface protein CD46 enables rapid screening for successful CRISPR-mediated knockin in human induced pluripotent stem cells (iPSCs). **a**. Boxplots depicting the HiBiT luminescence signal distribution before and after subcloning of CRISPR-targeted cells in the indicated wells. The dashed red line marks the background signal threshold, chosen based on initial standard curve measurements using recombinant HiBiT protein (not shown). **b**. Capillary electrophoresis following PCR amplification of the targeted CD46 region in non-targeted control (NC) iPSCs and four HiBiT/V5-targeted iPSC populations prior to subcloning. While a clear band-shift can be resolved in targeted cells, the resolution is insufficient to distinguish between a V5 (+42 bp) *vs* a HiBiT (+32 bp) knock-in. **c**. Example light micrographs of iPSC colonies following one round of subcloning, revealing healthy colony morphology with clearly defined edges. Scale bar = 1000 *µ*m. **d**. Summary of the CRISPR/Cas9 HiBiT-based targeting approach. Note that a second round of subcloning may be required depending on the initial targeting efficiency, in line with the original sib-selection approach ^4^. R, RevitaCell; RNPs, ribonucleoproteins; ssODN, single-stranded oligo DNA.

We report preliminary work seeking to develop an efficient and highly sensitive high-throughput method for detection of successful CRISPR-mediated knock-in in human iPSCs. To do this, we co-targeted the cell surface protein CD46 with a HiBiT peptide and a V5 tag, followed by luminescence-based screening of live cells to identify cells with successful editing of at least one allele. The results are summarised in Figure 1.

Prior to subcloning, the luminescence signal across 48 wells with targeted cells exhibited a relatively broad distribution, with multiple wells reaching a signal just above background (set to 5000 light units) ( Figure 1a). Only two had a signal between 10,000 and 15,000 light units. Cells from four wells with signal above 8000 light units were expanded, and two of these populations were subsequently used for subcloning into 96-well plates to enrich for HiBiT-positive cells. Within < 30 minutes, without the need for DNA extraction and Sanger sequencing of two 96-well plates, we were able to identify multiple wells with a substantial increase in luminescence signal relative to the overall population ( Figure 1), indicative of successful enrichment. Importantly, we continued to use a stringent background threshold to limit the number of false positives.

PCR-based amplification of the targeted CD46 region in the original four wells chosen for expansion was used to visualise the presence of a longer product ( Figure 1b). While neither method has the capacity to confirm whether or not the V5 tag has been incorporated into the second allele, the successful expression of HiBiT on the cell surface strongly suggests that at least one allele was targeted correctly in a subset of the examined cells.

The extracellular luminescence detection in live cells was compatible with pluripotent stem cell survival, with subcloned cells retaining a healthy colony appearance upon outgrowth ( Figure 1c). Thus, the enriched cells can be used for subsequent replica-plating and immunofluorescent detection of V5 with high-content imaging systems, substantially reducing expenses by only limiting detection to a few wells of a 96-well plate. A graphic summary of the overall CRISPR/Cas9 HiBiT-based targeting approach is provided in Figure 1d.

## Discussion

CRISPR-mediated knock-in in human iPSCs remains a challenge due to low efficiency, poor cell survival and the resulting need to screen a large number of clones. Combined with the high cost of human iPSC maintenance, relevant disease models based on this cell system are prohibitively expensive to many academic labs. We report a novel targeting strategy that utilises the HiBiT luminescence system to enable efficient screening for successfully CRISPR-edited iPSCs. The protocol uses live cells, thus eliminating the need for replica-plating, and can be executed within 30 minutes without compromising cell health.

Prior to applying this system, a standard curve should be generated using recombinant HiBiT-tagged protein in conditions mimicking the final cell-based assay in order to estimate background levels. Although background levels may differ due to different kit lots and/or cell densities, keeping a conservative luminescence threshold across experiments is recommended to limit false-positive hits. It is notable that the luminescence signal distribution prior to subcloning was broad, but centred around the background threshold, which might reflect the high sensitivity of the HiBiT system whereby the presence of rare cells with successful CRISPR-mediated knock-in of the tag can be picked up in multiple wells. It is important that future studies seeking to use this approach determine its compatibility as “co-selection” marker when the second targeting locus is distinct from CD46. It also remains to be determined whether CD46 HiBiT-tagging will be equally efficient in other iPSC lines.

We note that this approach does not provide a quantitative measure of knock-in efficiency. The actual efficiency may be low overall, with detection in the current format only possible due to the high sensitivity of the HiBiT system. Although we were able to distinguish a CD46 band shift by PCR across the targeted region, detection of luminescence has the advantage of confirming that the tagged protein is processed and expressed normally. This is a substantial improvement compared to conventional approaches based on laborious sequencing of a large number of clones to rule out unwanted indels in a CRISPR-targeted cell population.

Finally, we wish to highlight that our original aim was to test the use of HiBiT as an efficient and sensitive marker for successful editing of at least one *CD46* allele. The V5 tag was co-introduced to allow additional immunofluorescence-based screening of HiBiT-positive wells by high-content imaging. We hypothesise that this will allow easier detection of cells with concomitant V5 knock-in into the second allele while reducing the need to stain entire plates. For logistical reasons, we have not been able to confirm this in our system, but hope that this co-targeting strategy will be developed further by other researchers with access to our preliminary data. The idea of enriching for successfully edited cells by using co-targeting with phenotypic selection is not new, and several groups have systematically demonstrated the feasibility of this approach in the past. Using two different human iPSC lines, Mitzefelt *et al*. showed that TALEN-mediated knock-in of a puromycin resistance cassette into the ‘safe-harbour’ AAVS1 locus, and subsequent puromycin selection, enriches for simultaneous CRISPR/Cas9-induced HDR edits with ssODNs against multiple loci of interest ^11^. Using a distinct but conceptually similar approach in non-iPSC cell lines, Agudelo *et al.* used CRISPR-induced HDR to engineer endogenous Na+/K+ ATPase resistance to ouabain, alongside co-targeting of specific loci of interest, followed by ouabain-based enrichment for co-edited cells ^12^.

Relative to these studies, our observations are preliminary and will benefit from downstream validation, including comparisons to ‘mock’-edited cells. We do believe, however, that the approach combines several of the advantages of the prior studies above without some of their caveats. Among the most important advantages are: 1) the use of ssODNs to append an 11-amino-acid HiBiT tag to a cell surface protein (CD46) with little functional importance in most cultured cells, thus obviating the risk associated with random plasmid integration or disruption of a functionally important protein like the Na ^+^/K ^+^ ATPase; 2) the ability to perform relatively inexpensive and rapid (< 30 minutes) live-cell screening for the presence of CRISPR-targeted cells, which could theoretically be performed as early as 24 hours after targeting; 3) selection in the absence of pharmacological treatments with potential off-target effects that could compromise iPSC health.

## Conclusions

The HiBiT luminescence system can be used as a powerful tool to screen for successfully CRISPR-edited human iPSCs in knock-in experiments. The method is fast, cost-effective and minimises stress-inducing cell manipulations. Our preliminary data encourage additional development of the protocol by researchers using CRISPR to knock in point mutations or smaller tags into iPSCs or other challenging cell types. The system is versatile and tagged cells will be compatible with a wide range of downstream assays, including intravital imaging.

## Data availability

### Underlying data

Open Science Framework: Luminescent peptide tagging for efficient screening of gene-edited human iPSCs. https://doi.org/10.17605/OSF.IO/F82YD ^10^

This project includes the following underlying data:

- 2018-11-22_CRISPR_RNP_CD46_tag_knockin_iPSCs_before_subcloning_Hyperladder_100bp.tif ( *Gel image, validation gel*)

*−* 2018 *− *12 *− *01 *− H*11 *C*3 *−p*54 *− *96 *well − *25 *cells*. *per*. *well − to − replica − plate − *40 *x − * 2. (Raw micrograph image, D03 *Figure* 1 *C*)

*− *2018 *− *12 *− *01 *− H*11 *D*3 *− p*54 *− *96 *well − *12.5 *cells*. *per*. *well − to − replica − plate − *40 *x − *1. *tif* (Raw micrograph image, D03 *Figure* 1 *C*)

- 2019-01-11_Cap_electrophoresis_results.pptx (Lab chip capillary electrophoresis results, with additional description)

- LabChip GX Ralitsa & Semple 9 DNA 1 k samples 11th Jan 2019.pdf (Lab Chip capillary electrophoresis raw output data, *Figure* 1 *B*

- R_plots_for_publication.nb.html *Rcodeunderling Figure* 1 *a*)

-Readme_LabChip_Cap_Electrophoresis_results.pptx (additional information or interpretation of *Figure* 1 *B*)

Data are available under the terms of the Creative Commons Attribution 4.0 International license (CC-BY 4.0).
