# Supplementary material for: Luminescent peptide tagging enables efficient screening for CRISPR-mediated knock-in in human induced pluripotent stem cells
Source: Wellcome Open Res. 2019 Jul 11;4:37. Originally published 2019 Feb 20. [Version 3] doi: 10.12688/wellcomeopenres.15119.3 (PMC6640264; doi:10.12688/wellcomeopenres.15119.3)
Supplement: Peer review history

# Peer review: Reviewer response for version 3

**DOI:** 10.21956/wellcomeopenres.16770.r35971

**Published:** 2019-07-18

**Authors:** Aron M. Geurts, Maribel Marquez

**Affiliations:** 1 Department of Physiology, Medical College of Wisconsin, Milwaukee, WI, USA

The schematic figure is very helpful, thank you. I have no further comments.

We confirm that we have read this submission and believe that we have an appropriate level of expertise to confirm that it is of an acceptable scientific standard.

# Peer review: Reviewer response for version 2

**DOI:** 10.21956/wellcomeopenres.16637.r35487

**Published:** 2019-06-10

**Authors:** Aron M. Geurts, Maribel Marquez

**Affiliations:** 1 Department of Physiology, Medical College of Wisconsin, Milwaukee, WI, USA

This is a clever and promising approach with promise to shorten the costly and laborious process associated with precise gene editing in iPSCs.

- I am not certain if there are restrictions on length or the number of references allowed, but there are several literature examples where similar 'co-targeting' methods have been developed to enrich targeting in cells (including iPSCs). In my opinion, these should be cited and compared/contrasted with this approach in the discussion. I cited examples below.
- Please describe DPBS and the source.
- Promising progress has been made demonstrating the ability to detect luminescence, however the results fall short of the minimum requirements demonstrating successful CRISPR-stimulated targeting. Sequence validation showing the intended proper homology-directed repair to insert either or both tags in clones is an essential missing piece of data.
- In the subcloning step from 48 to 96 wells, clarify whether this was single-cell subcloning. Is it expected that the analysis at this stage is measuring luminescence in single clones?
- The gross appearance of cells looks promising following targeting. However, this also does not meet the minimum standards of publication in my opinion. At a minimum, a pluripotency marker analysis (Oct4, Sox2, Nanog TRA-1-60) should be demonstrated to be unchanged in edited clones following targeting and screening. The images presented are also dark and are not compared to un-treated cells.
- The claim that targeted clones can be identified in 30 minutes is somewhat misleading. The researcher has multiple screening steps and in the end must sequence validate the target of interest. I agree, however, that this approach would likely save days of labor and hundreds of dollars of unnecessary sequencing. A figure depicting the strategy and timeline from transfection to subcloning to identification of properly edited clones would be helpful. 

We confirm that we have read this submission and believe that we have an appropriate level of expertise to confirm that it is of an acceptable scientific standard, however we have significant reservations, as outlined above.

## References

[1] : Marker-free coselection for CRISPR-driven genome editing in human cells. *Nat Methods*.2017;14(6) : 10.1038/nmeth.4265 615-620 10.1038/nmeth.4265 28417998

[2] : Efficient Precision Genome Editing in iPSCs via Genetic Co-targeting with Selection. *Stem Cell Reports*.2017;8(3) : 10.1016/j.stemcr.2017.01.021 491-499 10.1016/j.stemcr.2017.01.021 PMC535564328238794

# Response

**Published:** 2019-07-02

**Authors:** Ralitsa Madsen

**Affiliations:** University of Edinburgh, UK

**Reviewer Comment:**

- I am not certain if there are restrictions on length or the number of references allowed, but there are several literature examples where similar 'co-targeting' methods have been developed to enrich targeting in cells (including iPSCs). In my opinion, these should be cited and compared/contrasted with this approach in the discussion. I cited examples below.

**Author Response:**

*We thank the reviewers for this suggestion and have now added the following to the discussion:*

*"The idea of enriching for successfully edited cells by using co-targeting with phenotypic selection is not new, and several groups have systematically demonstrated the feasibility of this approach in the past. Using two different human iPSC lines, Mitzefelt et al. showed that TALEN-mediated knock-in of a puromycin resistance cassette into the ‘safe-harbour’ AAVS1 locus, and subsequent puromycin selection, enriches for simultaneous CRISPR/Cas9-induced HDR edits with ssODNs against multiple loci of interest. Using a distinct but conceptually similar approach in non-iPSC cell lines, Agudelo et al. used CRISPR-induced HDR to engineer endogenous Na ^+^/K ^+^ATPase resistance to ouabain, alongside co-targeting of specific loci of interest, followed by ouabain-based enrichment for co-edited cells.*

*Relative to these studies, our observations are preliminary and will benefit from downstream validation, including comparisons to ‘mock’-edited cells. We do believe, however, that the approach combines several of the advantages of the prior studies above without some of their caveats. Among the most important advantages are: 1) the use of ssODNs to append an 11-amino-acid HiBiT tag to a cell surface protein (CD46) with little functional importance in most cultured cells, thus obviating the risk associated with random plasmid integration or disruption of a functionally important protein like the Na ^+^/K ^+^ATPase*; 2) *the ability to perform relatively inexpensive and rapid (< 30 minutes) live-cell screening for the presence of CRISPR-targeted cells, which could theoretically be performed as early as 24 hours after targeting; 3) selection in the absence of pharmacological treatments with potential off-target effects that could compromise iPSC health."* 

   

**Reviewer Comment:**

- Please describe DPBS and the source.

**Author Response:**

*Dulbecco’s Phosphate Buffered Saline (no calcium, no magnesium; Thermo Fisher Scientific catalogue no: 14190144) has now been added to the Methods section.*

- Promising progress has been made demonstrating the ability to detect luminescence, however the results fall short of the minimum requirements demonstrating successful CRISPR-stimulated targeting. Sequence validation showing the intended proper homology-directed repair to insert either or both tags in clones is an essential missing piece of data.

**Author Response:**

*We acknowledge that our study is limited, hence our reporting it as a research note, in the hope that others working in this area may develop it further.*

**Reviewer Comment:**

- In the subcloning step from 48 to 96 wells, clarify whether this was single-cell subcloning. Is it expected that the analysis at this stage is measuring luminescence in single clones?

**Author Response:**

*We believe this is specified in the following methods paragraph, but would be happy to comment further on this if required. We also wish to highlight that we find this approach to be advantageous to single colony-picking as it is less harsh to the cells and results in survival of 2-3 cells, thus limiting the confounding effects that may arise from the potential enrichment of abnormal clones exposed to single cell bottleneck selection.*

*“Following recovery for 1-2 days, cells with a positive luminescence signal were expanded non-enzymatically (using ReLeSR) into a Geltrex-coated 24-well plate, prior to a round of subcloning into Geltrex-coated 96-well plates, seeding 12.5 cells/well in E8/F supplemented with 1X RevitaCell. From previous work, we know that this seeding density typically results in survival of 2-3 cells in each well and allows for efficient enrichment while minimising cell loss* *^6^* *“*

**Reviewer Comment:**

- The gross appearance of cells looks promising following targeting. However, this also does not meet the minimum standards of publication in my opinion. At a minimum, a pluripotency marker analysis (Oct4, Sox2, Nanog TRA-1-60) should be demonstrated to be unchanged in edited clones following targeting and screening. The images presented are also dark and are not compared to un-treated cells.

**Author Response:**

*We agree that further validation is required as suggested. This is beyond the scope of this research note. However, we have included additional light microscopy images of colonies from each plate with subcloned cells (originating from each one of the four independent hit wells B03, C03, D03, E06); in addition to the images added to Figure 1c, others have been included in the accompanying Open Science Framework project (DOI 10.17605/OSF.IO/F82YD). We have attempted to increase the contrast of images in the main figure. Better quality light microscopy images were difficult to obtain at this stage as each single cell in the original well tended to crawl to the edge, which made subsequent imaging of the growing colonies difficult at high resolution.*

**Reviewer Comment:**

- The claim that targeted clones can be identified in 30 minutes is somewhat misleading. The researcher has multiple screening steps and in the end must sequence validate the target of interest. I agree, however, that this approach would likely save days of labor and hundreds of dollars of unnecessary sequencing. A figure depicting the strategy and timeline from transfection to subcloning to identification of properly edited clones would be helpful. 

**Author Response:**

*We believe that we have specified that the 30 minutes apply to the initial screening step and not to the extensive validation steps which will be required once potential hits have been specified. We would therefore disagree with the reviewer that our statement is misleading in this context.*

*We think a figure is a great suggestion and have included a schematic outline in the main figure.*

# Peer review: Reviewer response for version 2

**DOI:** 10.21956/wellcomeopenres.16637.r35320

**Published:** 2019-05-13

**Authors:** Sara E. Howden

**Affiliations:** 1 Murdoch Children's Research Institute (MCRI), Parkville, Victoria, Australia

Approved.

I confirm that I have read this submission and believe that I have an appropriate level of expertise to confirm that it is of an acceptable scientific standard.

# Peer review: Reviewer response for version 1

**DOI:** 10.21956/wellcomeopenres.16495.r34940

**Published:** 2019-03-13

**Authors:** Sara E. Howden

**Affiliations:** 1 Murdoch Children's Research Institute (MCRI), Parkville, Victoria, Australia

In this study, Madsen and colleagues describe the use of HiBiT technology to tag the surface membrane protein CD46 in human pluripotent stem cells, in order to identify successfully gene-edited cells using luminescence detection in live cells. The authors claim that this makes gene editing in human iPSCs less costly because this approach substantially reduces labour-intensive screening of CRISPR-targeted iPSCs and minimises the number of subcloning steps.

- The authors should explain in more detail the purpose of the V5 tag. I am guessing that the purpose of this study is to use the HiBiT tag as a way to identify cells that have also incorporated the V5 tag on the second allele? If this is the case, this should be specifically stated. Perhaps it would be helpful if there was a diagram depicting the targeting strategy.
- How many wells of a 96-well plate are cells plated into following transfection? The authors state that “Only two wells had a signal between 10,000 and 15,000 light units”. Two out of how many? Please state as %.
- Is there a way to identify and specifically mark individual colonies within a well that have successfully incorporated the HiBiT tag? These could then theoretically be isolated by picking and expanding, thereby reducing subsequent rounds of subcloning.
- Lastly, although the authors claim this work is preliminary, it would be nice to see this particular study a little more complete. Of particular interest would be whether a pure population of cells harbouring both tags could in fact be obtained, how many rounds of subcloning are necessary to obtain this, and whether the cells maintain genomic integrity at the completion of this process.

I confirm that I have read this submission and believe that I have an appropriate level of expertise to confirm that it is of an acceptable scientific standard, however I have significant reservations, as outlined above.

# Response

**Published:** 2019-04-09

**Authors:** Ralitsa Madsen

**Affiliations:** University of Edinburgh, UK

We thank Dr Howden for her review of our Research Note on the use of a luminescence peptide for efficient identification of iPSCs undergoing successful CRISPR-mediated knock-in. 

We have addressed Dr Howden's reservations as follows: 

***The authors should explain in more detail the purpose of the V5 tag. I am guessing that the purpose of this study is to use the HiBiT tag as a way to identify cells that have also incorporated the V5 tag on the second allele? If this is the case, this should be specifically stated. Perhaps it would be helpful if there was a diagram depicting the targeting strategy.***

**

*We agree with Dr Howden that this needs further clarification. The following paragraph has now been added to the Discussion section of the revised manuscript:*

*"Finally, we wish to highlight that our original aim was to test the use of HiBiT as an efficient and sensitive marker for successful editing of at least one CD46 allele. The V5 tag was co-introduced to allow additional immunofluorescence-based screening of HiBiT-positive wells by high-content imaging. We hypothesise that this will allow easier detection of cells with concomitant V5 knock-in into the second allele while reducing the need to stain entire plates. However, for logistical reasons, we have not been able to confirm this ourselves, but hope that this co-targeting strategy will be developed further by other researchers with access to our preliminary data."*

**How many wells of a 96-well plate are cells plated into following transfection? The authors state that “Only two wells had a signal between 10,000 and 15,000 light units”. Two out of how many? Please state as %.**

*A total of 48 wells of a 96-well plate were seeded following nucleofection of the iPSC suspension. This has now been added to the results section of the revised manuscript. However, we wish to refrain from “%” measures as this might wrongly be interpreted as a measure of the knock-in efficiency. As outlined in the Discussion section, this method does not provide a quantitative measure of knock-in efficiency which may be very low overall and the knock-in is only possible to detect due to the high sensitivity of HiBiT-based luminescence detection.*

**Is there a way to identify and specifically mark individual colonies within a well that have successfully incorporated the HiBiT tag? These could then theoretically be isolated by picking and expanding, thereby reducing subsequent rounds of subcloning.**

*This is not necessary with the sib-selection method that we are using. Its purpose is to avoid manual colony picking as described in the original paper on this method (Miyaoka et al. Nat Methods 2014). We have optimised the subcloning process for our iPSC line so that each well contains less than 5 colonies (the majority contain 2-3) and have previously used this method for rapid enrichment of a homogenous population of iPSCs with a knock-in modification of interest (Madsen et al. PNAS 2019). The extent of subcloning and the number of subcloning rounds will, however, depend on the starting knock-in efficiency and the sensitivity of the post-CRISPR screening method. This is why we chose HiBiT-based luminescence as a highly sensitive screening strategy, particularly in cases where the knock-in efficiency might be low.*

**Lastly, although the authors claim this work is preliminary, it would be nice to see this particular study a little more complete. Of particular interest would be whether a pure population of cells harbouring both tags could in fact be obtained, how many rounds of subcloning are necessary to obtain this, and whether the cells maintain genomic integrity at the completion of this process.**

*We agree with Dr Howden that further development of the method is needed. However, logistical hurdles have prevented us from doing this ourselves. Our hope is that this Research Note will be useful to others who might wish to improve the method and test its utility as co-targeting strategy. This has now been stated in the Discussion.*
